# Evaluation of an Extended Stroke Rehabilitation Service (EXTRAS): A Randomized Controlled Trial and Economic Analysis

**DOI:** 10.1161/STROKEAHA.119.024876

**Published:** 2019-10-22

**Authors:** Helen Rodgers, Denise Howel, Nawaraj Bhattarai, Robin Cant, Avril Drummond, Gary A. Ford, Anne Forster, Richard Francis, Katie Hills, Anne-Marie Laverty, Christopher McKevitt, Peter McMeekin, Christopher I.M. Price, Elaine Stamp, Eleanor Stevens, Luke Vale, Lisa Shaw

**Affiliations:** 1 From the Stroke Research Group, Institute of Neuroscience (H.R., G.A.F., R.F., K.H., C.I.M.P., L.S.), Newcastle University, Newcastle upon Tyne, United Kingdom; 2 Institute of Health and Society Newcastle University, Newcastle upon Tyne, UK (D.H., N.B., E. Stamp, L.V.), Newcastle University, Newcastle upon Tyne, United Kingdom; 3 Lay Investigator, Stroke Research Group, Institute of Neuroscience, Newcastle (R.C.), Newcastle University, Newcastle upon Tyne, United Kingdom; 4 Stroke Northumbria, Northumbria Healthcare NHS Foundation Trust, North Tyneside, United Kingdom (H.R., A.-M.L., C.I.M.P.); 5 Newcastle Hospitals NHS Foundation Trust, Newcastle upon Tyne, United Kingdom (H.R.); 6 School of Health Sciences, Nottingham University, United Kingdom (A.D.); 7 Medical Sciences Division, University of Oxford, and Oxford University Hospitals NHS Foundation Trust, United Kingdom (G.A.F.); 8 Academic Unit of Elderly Care and Rehabilitation, University of Leeds, United Kingdom (A.F.); 9 School of Population Health and Environmental Sciences, King’s College London, United Kingdom (C.M., E. Stevens); 10 Faculty of Health and Life Sciences, Northumbria University, Newcastle upon Tyne, United Kingdom (P.M.).

**Keywords:** activities of daily living, goals, quality-adjusted life years, stroke rehabilitation, survivors

## Abstract

There is limited evidence to guide rehabilitation to meet the longer term needs of stroke survivors. The clinical effectiveness and cost-effectiveness of an extended stroke rehabilitation service (EXTRAS) provided following early supported discharge were determined.

**Methods—:**

EXTRAS was a pragmatic parallel-group observer-blind randomized controlled trial involving 19 UK centers. Patients with stroke were individually randomized to receive EXTRAS or usual care at discharge from early supported discharge. Five EXTRAS reviews were provided by an early supported discharge team member between one and 18 months, usually by telephone. Reviews consisted of a semi-structured interview assessing progress, rehabilitation needs, and service provision, with goal setting and action planning. The primary outcome was performance in extended activities of daily living (Nottingham EADL Scale) at 24 months post-randomization. The Nottingham EADL Scale is scored 0 to 66, with higher scores indicating better performance in these activities. Cost-effectiveness was estimated using resource utilization costs and Quality Adjusted Life Years. Analyses were intention to treat.

**Results—:**

Between January 9, 2013 and October 26, 2015, 573 participants were randomized (EXTRAS, n=285; usual care, n=288). Mean 24 month Nottingham EADL Scale scores were EXTRAS (n=219) 40.0 (SD 18.1) and usual care (n=231) 37.2 (SD 18.5) giving an adjusted mean difference of 1.8 (95% CI, –0.7 to 4.2). 1155/1338 (86%) of expected EXTRAS reviews were undertaken. Over 24 months, the mean cost of resource utilization was lower in the intervention group: –£311 (–$450 [95% CI, −£3292 to £2787; −$4764 to $4033]). EXTRAS provided more Quality Adjusted Life Years (0.07 [95% CI, 0.01 to 0.12]). At current conventional thresholds of willingness to pay (£20 000 [$28 940] per Quality Adjusted Life Years), there was a 90% chance that EXTRAS could be considered cost-effective.

**Conclusions—:**

EXTRAS did not significantly improve stroke survivors’ performance in extended activities of daily living. However, given the impact on costs and Quality Adjusted Life Years, EXTRAS may be an affordable addition to improve stroke care.

**Clinical Trial Registration—:**

URL: www.isrctn.com. Unique identifier: ISRCTN45203373.

## Abstract 2

Supplemental Digital Content is available in the text.

Around two-thirds of stroke, survivors leave hospital with disability. Some make a full recovery while for others stroke is a long-term condition. Over 1.2 million stroke survivors live in the United Kingdom where stroke is the commonest cause of complex disability.^1^

Stroke units and early supported discharge (ESD) services have been shown to be clinically effective and cost-effective.^2,3^ However, there is limited evidence to guide rehabilitation to optimize recovery and meet the longer term needs of stroke survivors. Lack of evidence of effectiveness has meant that provision of longer term stroke care remains a low priority for those who are responsible for funding the development of new services and as a result service development has lagged behind developments for acute care and early rehabilitation. Provision of longer term services beyond ESD are limited and vary across the United Kingdom,^4,5^ and many stroke survivors feel marginalized in the longer term by health and social services.^6^ This study evaluated a new longer term community stroke rehabilitation service which commenced when routine ESD ended.

## Methods

### Study Design

The methods of the extended stroke rehabilitation service (EXTRAS) trial have been reported previously.^7^ The study was a parallel-group observer-blind multicenter individually randomized controlled trial which took place in 19 UK National Health Service (NHS) study centers. All study centers had an ESD service comprising a multidisciplinary stroke team which provided rehabilitation in the community, commencing within 48 hours of discharge from hospital. Ethical approval was granted by the National Research Ethics Committee.

We will make anonymized data from this research project available to the scientific community with as few restrictions as feasible, while retaining exclusive use until the publication of major outputs. Those interested in accessing data should contact the corresponding author.

### Participants

Adults with a new stroke (first-ever or recurrent) were eligible to take part if they received ESD and were able to participate in a rehabilitation program focussing on extended activities of daily living. Participants provided written consent. Aphasia friendly study materials were available for patients with communication difficulties. Patients lacking capacity to consent could be included if a consultee agreed to their enrollment and was prepared to assist with study processes.

### Randomization and Blinding

Randomization to a study group took place at discharge from routine ESD using an online web-based service. Stratification by study center was used, and permuted block sequences assigned participants to intervention or control in a 1:1 ratio. Due to the nature of the intervention, patients, carers, and staff providing EXTRAS could not be blinded to study group. However, outcome data were intended to be collected by a blinded researcher, and any unblinding was recorded.

### Intervention

The extended stroke rehabilitation service sought to help individuals to maximize their recovery and adjust to their residual disability in the context of their day to day activities. A senior member of the ESD team provided 5 structured reviews at 1, 3, 6, 12, and 18 months post-ESD. Most reviews were intended to be conducted by telephone and covered issues identified in a national survey of patient needs^8^ and other literature: mobility; personal care; mealtimes; domestic activities; work and volunteering; hobbies and interests; driving and transport; communication; memory and concentration; mood, anxiety and depression; medical issues; pain; and other issues. The patient’s progress was reviewed, along with their current rehabilitation needs and service provision. Agreed rehabilitation goals were set, and an action plan was made at each review. The action plan could include verbal advice and encouragement; discussion with services currently involved in the patient’s care; signposting to local activities, community services or voluntary services; referral to stroke services, rehabilitation services or primary care. The reviews were multifaceted, reflecting current clinical practice, and included self-management and feedback. EXTRAS was provided in addition to usual NHS care. The number of interviews and their intervals was a pragmatic decision based on what would be achievable and affordable within the NHS. A detailed description of EXTRAS using the Template for Intervention Description and Replication checklist^9^ is provided in Table I in the online-only Data Supplement.

### Control

Patients randomized to the control group received usual NHS care. After completion of routine ESD, patients who had ongoing rehabilitation needs could be referred to a range of services (eg, neurorehabilitation teams; day hospital; community rehabilitation services) depending on local availability.

### Data Collection and Outcome Measures

Before randomization, baseline data collection included: demography; stroke type and subtype; performance in extended activities of daily living (Nottingham Extended Activities of Daily Living [NEADL] Scale)^10^; health status (Oxford Handicap Scale [OHS])^11^; mood (Hospital Anxiety and Depression [HAD] Scale)^12^; quality of life (EQ-5D-5L)^13^; and prestroke resource usage (adaption of Client Service Receipt Inventory ).^14^

Outcome data were collected by telephone at 12 and 24 months post-randomization by a researcher based in the study co-ordinating center. Where a telephone interview was not possible, a postal questionnaire or a face to face assessment was undertaken.

The primary outcome was performance in extended activities of daily living measured by the NEADL Scale^10^ at 24 months post-randomization. Secondary outcomes were health status (OHS),^11^ mood (HAD Scale),^12^ and experience of services (survey based on Picker Institute questions)^15^ at 12 and 24 months post-randomization. Quality-adjusted life-years (QALYs) were derived from responses to the EQ-5D-5L^13^ and an England population tariff.^16^ Resource utilization data (adaption of Client Service Receipt Inventory)^14^ were used to generate an average cost per participant. Questions to capture adverse events were included in study outcome proformas.

### Statistical Methods

To provide 90% power to detect a clinically important difference of 6 points on the NEADL Scale^10^ (scored 0–66, SD 18), follow-up data from 382 patients split equally between intervention, and control groups were required. Based on other stroke rehabilitation studies, it was estimated that there could be up to 25% attrition between randomization and 24 months. To allow for this, 510 patients were required to be randomized into the study.

Analyses were performed on an intention to treat basis. Where no more than 20% of questions were missing data on specific scales, simple imputation was used to replace missing items.^17^ Mean scores on the NEADL Scale were compared between intervention and control groups using multiple linear regression, including terms for center, baseline OHS, age and sex. In terms of secondary outcomes, ordinal regression was used to analyze the OHS, and multiple linear regression was used to analyze the 2 separate domains of the HAD Scale, all analyses were adjusted for the same covariates as above. The responses to the individual experience of service questions were dichotomized to create proportions of patients “in agreement” or “satisfied,” and the difference in proportions between groups reported with a 95% CI. In addition, a post hoc analysis of the HAD Scale considered those patients scoring 8 or more to be cases of anxiety or depression as this is how the HAD Scale is used in clinical practice,^12^ and logistic regression was used to compare the randomization groups on this binary variable at 12 and 24 months. Odds ratios with 95% CI were reported for an unadjusted model, and one adjusted for the covariates listed above.

Cost-effectiveness was estimated from a health and social care providers’ perspective over the trial period. Unit costs for resources used were obtained from routine sources, for example, Personal Social Services Research Unit and NHS Reference costs.^18^ For EXTRAS, each review was estimated to require 2 hours of staff time, costed at £44 ($64) per hour. Costs in the second year were discounted (3.5%) giving a total EXTRAS cost of £437 ($632) per patient. Differences in costs were estimated using a general linear model with a gamma distribution adjusting for center, baseline costs, age and sex. In terms of QALYs, linear regression was used to compare intervention and control groups adjusting for center, baseline utility, age and sex. A cost-effectiveness plane was constructed using QALY data.

## Results

**Figure 1.**
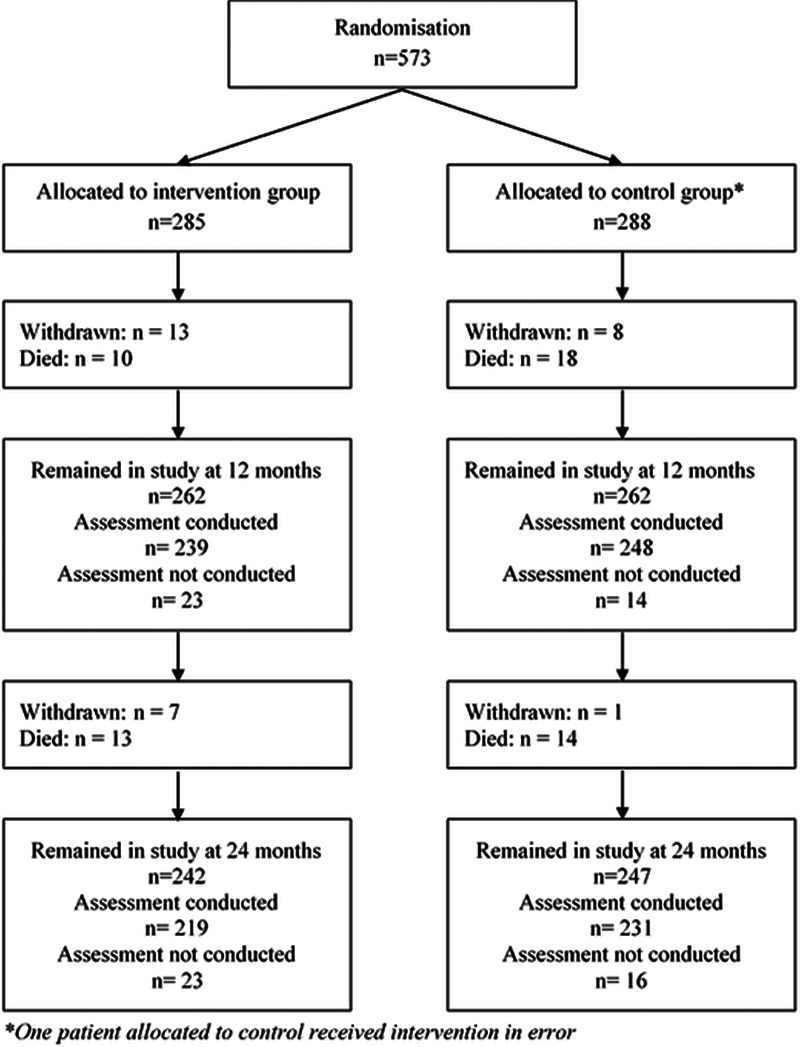
Trial profile.

Between January 09, 2013 and October 26, 2015, 573 patients were randomized (intervention group n=285, control group n=288). Retention and follow-up are shown in Figure 1. At 24 months, outcome data were available for 450/573 (78.5%) patients. Fifty-five (9.6%) participants died within the study period, and 29 (5.1%) withdrew from the study.

Baseline demography and stroke characteristics are shown in Table 1. The randomization groups were well matched. The median time between stroke and randomization was 72 days.

**Table 1.**
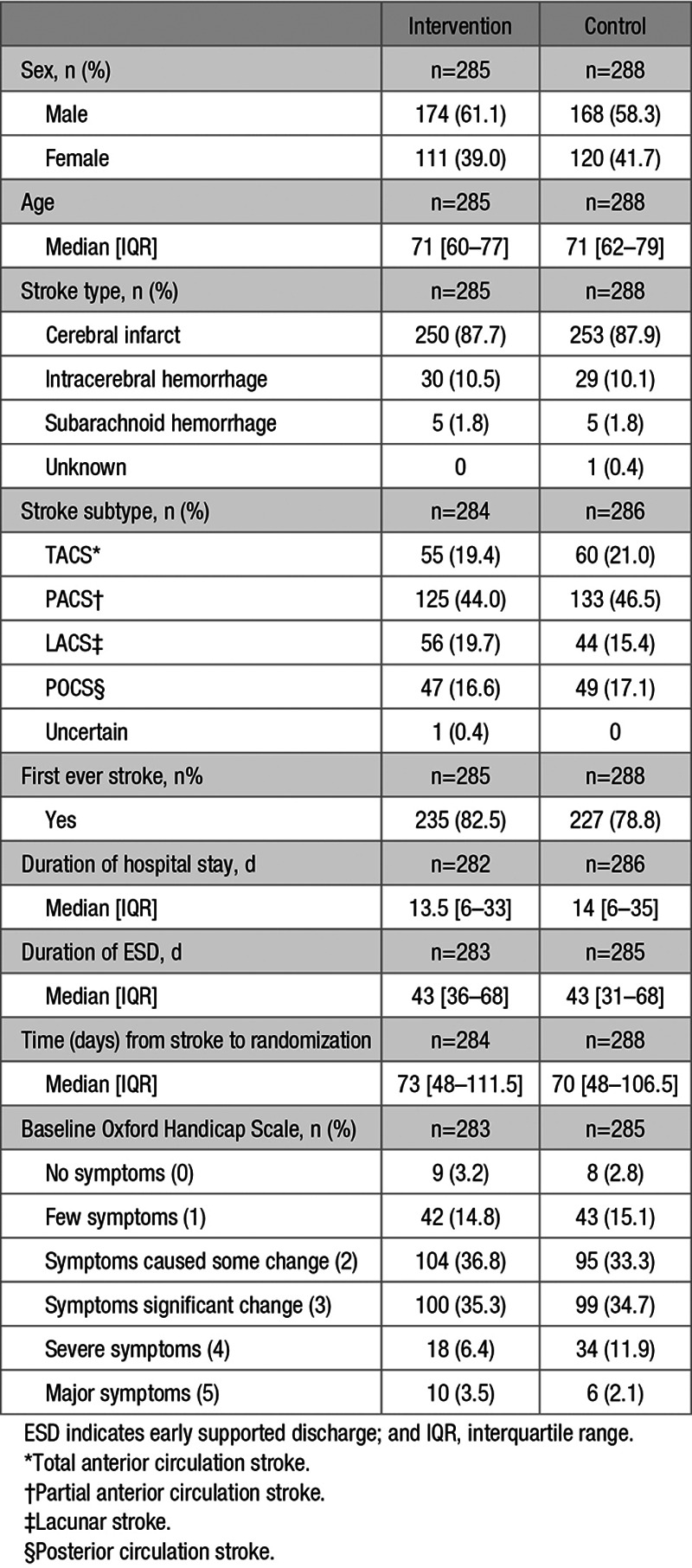
Demography and Stroke Characteristics

Adherence to delivery of the EXTRAS reviews was high: 1155/1338 (86%) of expected reviews were completed with 914/1155 (79%) being undertaken by telephone. A physiotherapist conducted 56% of reviews. Other professionals involved were occupational therapists (28%), nurses (8%), speech and language therapists (5%), stroke co-ordinators (1%), and dieticians (1%). Of the 258 patients who received at least 2 reviews, 167 (65%) had all of their reviews conducted by the same reviewer. Full details of the content of EXTRAS reviews will be reported elsewhere.

The mean NEADL Scale scores at prestroke, baseline, 12 and 24 months are shown in Table 2. Patients reported that they undertook most activities before their stroke, but the mean scores at baseline decreased to 39.8 (SD 16.1) in the intervention group and 39.1 (SD 16.1) in the control group. Thereafter, the mean scores remained very similar over time for those patients remaining in the intervention group and decreased very slightly in the control group. The adjusted difference in means at 24 months (primary outcome analysis) was 1.8 (95% CI, −0.7 to 4.2). As the minimum clinically important difference on the NEADL Scale is 6, the results were not consistent with a meaningful change on this scale.

**Figure 2.**
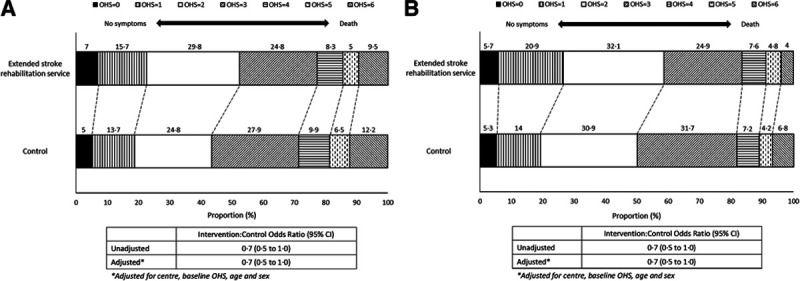
Distribution of Oxford Handicap Scale (OHS) scores. **A**, At 12 mo. **B**, At 24 mo.

Figure 2 shows the distribution of OHS scores at 12 and 24 months. At 24 months, the odds of the intervention group being in worse health was 0.7× as high than for control patients (95% CI, 0.5 to 1.0). The comparison at 12 months was similar.

The mean anxiety and depression HAD Scale scores were lower for those in the intervention group at 12 and 24 months, but the 95% CI for the difference in scores included zero. However, the post hoc caseness analysis found that in the intervention group, there were significantly fewer cases of depression at 12 months (29% intervention group versus 40% control group) and significantly fewer cases of anxiety at 24 months (28% intervention group versus 38% control group; Table 2).

**Table 2.**
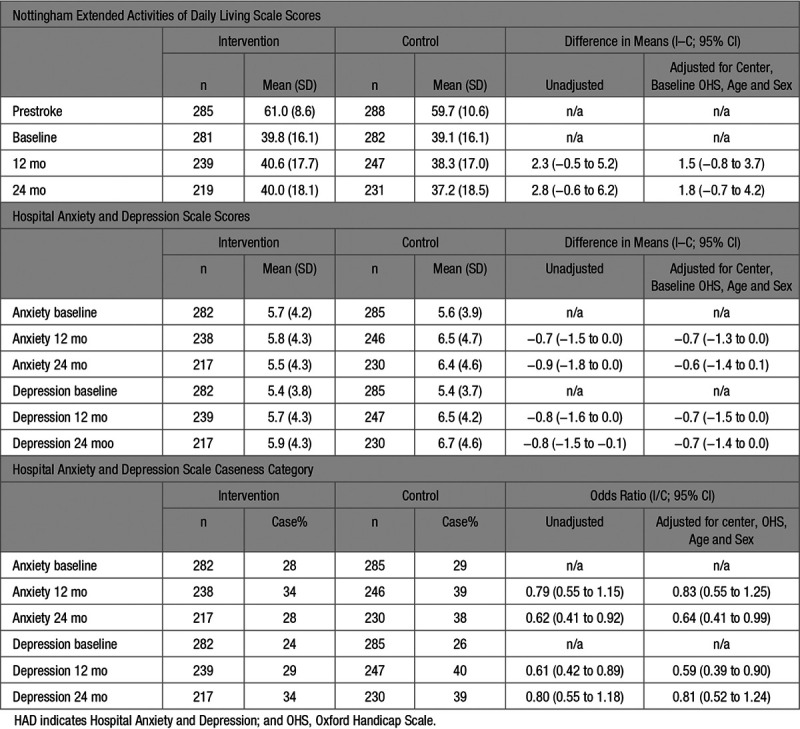
Comparison of Performance in Nottingham Extended Activities of Daily Living (NEADL Scale) and Mood (HAD Scale)

For 4of the 19 aspects of care examined in the experience of services survey at 24 months, the 95% CI for the differences in percentage “in agreement” or “satisfied” between the groups did not cover the value zero. These were “staff treated you with dignity and respect,” “staff met your needs,” “overall satisfaction,” and “help with mobility.” These data indicate that patients in the intervention group appeared to be more satisfied with these aspects of care (Table II in the online-only Data Supplement).

**Figure 3.**
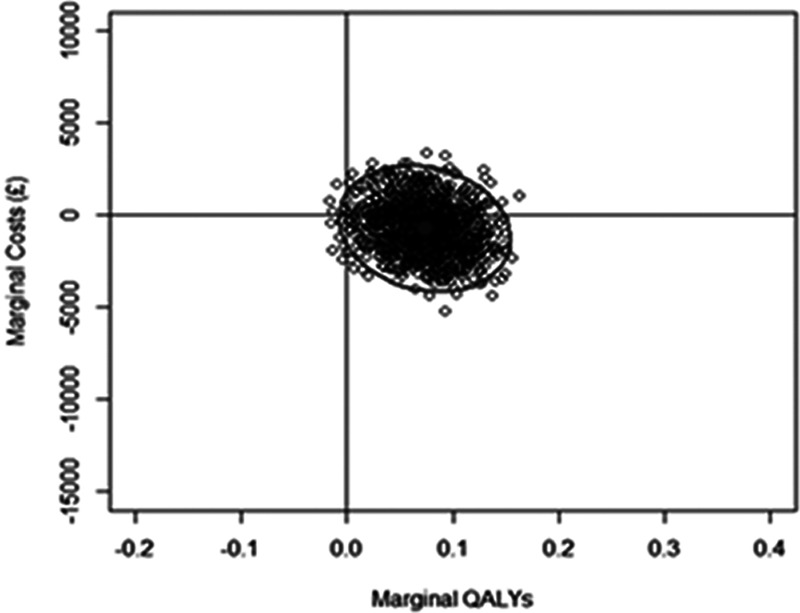
Cost-effectiveness plane. QALYs indicates quality-adjusted life-years.

Differences in EQ-5D-5L utilities, QALYs and costs are shown in Table 3. UK Sterling (£) were converted to US Dollars ($) using the purchasing power parities rates for 2017 (£1=USD 1.447).^19^ Patients in the intervention group experienced 0.07 (95% CI, 0.01 to 0.12) additional QALYs. The mean cost of resource utilization was lower in the intervention group: −£311 (−$450 [95% CI, −£3292 to £2787; −$4764 to $4033). Cost savings were predominantly in social care rather than health care. Figure 3 shows the cost-effectiveness plane and demonstrates that there is a 68% chance that EXTRAS is cost saving. At the current NHS standard of willingness to pay £20 000 ($28 940) per QALY, there was a 90% probability that the EXTRAS intervention is cost-effective.

**Table 3.**
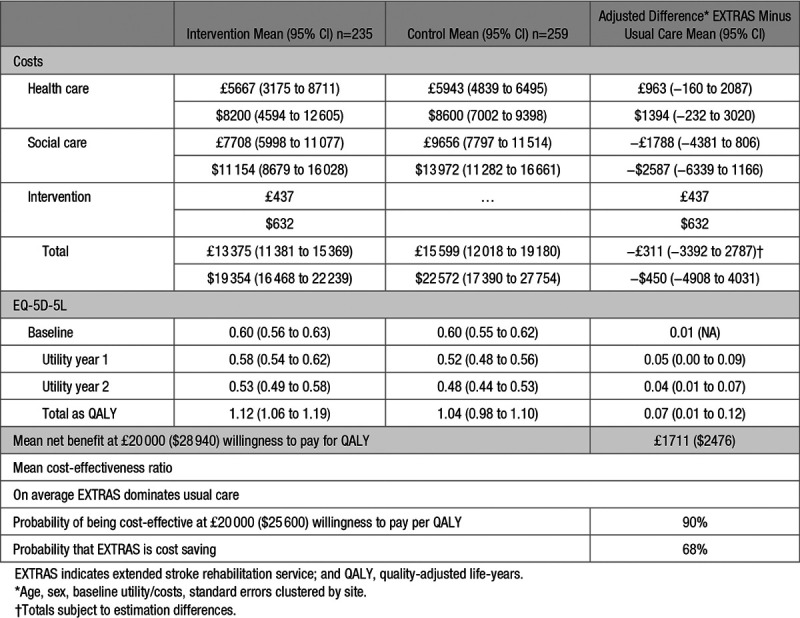
Costs, EQ-5D-5L Utilities, and QALYs

No association was found between the effectiveness of the intervention and prestroke OHS score, baseline NEADL Scale score and time in organized stroke care (defined as time as an inpatient and time in ESD) which were prespecified exploratory analyses (Figures IA through IC in the online-only Data Supplement).

At 12 months, telephone or face to face outcome assessments were conducted for 212 patients in the intervention group and 204 in the control group. The remainder were undertaken by post. The outcome assessor reported that they were unmasked for 37/212 (17.4%) intervention group participants and 7/204 (3.4%) in the control group. At 24 months, these figures were 17/195 (8.7%) and 2/186 (1.1%) respectively.

Serious adverse events were reported for 125/285 (43.9%) patients in the intervention group (total 250 events) and 130/288 (45.1%) patients in the control group (total 254 events). There were no significant differences in number of events per patient between the randomization groups.

## Discussion

The extended stroke rehabilitation service (EXTRAS) did not improve stroke patients’ performance in extended activities of daily living when compared with usual care. However, there were fewer cases of anxiety and depression in the intervention group, and patients in the intervention group were more satisfied with some aspects of their care. Although there is no evidence of a treatment effect on the primary outcome, the cost-effectiveness analyses are consistent with EXTRAS being associated with cost saving and health-related quality of life gain. One explanation of this finding is that the EQ-5D-5L captured wider impacts of EXTRAS than the NEADL Scale. The Nottingham EADL scale measures performance in extended activities of daily living, for example, mobility, household chores. The EQ-5D-5L is broader covering: mobility, self-care, usual activities, pain/discomfort, and anxiety/depression.

Divergent results for primary outcome and health economic analyses have been reported in other settings,^20^ but we are not aware of any stroke rehabilitation trials which have reported this combination of findings. In clinical trials, the primary outcome is chosen as it is believed to be the outcome of greatest importance. Our intervention sought to improve performance in activities of daily living, and it did not. Secondary outcomes are used to evaluate additional effects of an intervention which may be related to the primary question or other hypotheses. These effects are also important, but secondary outcome results in the context of a neutral primary outcome are generally regarded as hypothesis-generating rather than hypothesis testing. Nevertheless, our findings suggest that the intervention may have improved health-related quality of life, mood, and aspects of satisfaction with services. In hindsight, performance in extended activities of daily living may not have been the best primary outcome measure for the EXTRAS trial as this was only one component of recovery after stroke which the intervention sought to improve. A more global measure of health-related quality of life or societal participation would have been a good alternative.

EXTRAS reviews offered stroke survivors regular preplanned contact with a stroke specialist, who was part of a multidisciplinary team, who knew about their stroke journey. Although this trial was undertaken in the United Kingdom, the EXTRAS intervention could be used in any healthcare setting which provides ESD. Stroke survivors had the opportunity to discuss their achievements and concerns and to set goals and agree action points for ongoing issues. The reviewer supported them to reflect on their situation and to be realistic about their progress and expectations as well as to become more knowledgeable about stroke and the help which was and was not available. Self-management was an important part of the intervention as we sought to improve the stroke survivor’s knowledge, skills, and confidence to manage their condition. It is, therefore, perhaps, not surprising that benefits were seen in terms of wellbeing, mood and satisfaction with services rather than physical activities.

A number of studies have identified that stroke survivors have a wide range of longer term physical, psychological, and social needs.^4,6,8^ Trials which evaluated interventions seeking to address either single or multiple needs have used a range of approaches and outcomes, and are of variable quality. Individual community-based rehabilitation studies and subsequent meta-analyses have been largely neutral across a range of interventions and outcomes. Few studies included a health economic evaluation, and QALYs have rarely been reported.^21,22^

When designing the EXTRAS trial in 2010, we were encouraged by the results of a 2003 Cochrane review (14 trials, 1617 participants) which reported that therapy-based services (excluding ESD services) provided to patients with stroke soon after discharge from hospital reduced the odds of a poor outcome (OR, 0.72 [95% CI, 0.57 to 0.92]) and increased performance in personal activities of daily living compared to usual care.^23^ However, a 2008 Cochrane review (5 trials, 487 participants) found inconclusive evidence that similar interventions provided one year or more after stroke were effective.^24^

Our intervention went beyond trying to improve performance in extended activities of daily living as we sought to address the full range of long-term needs experienced by stroke survivors, and therefore EXTRAS has some similarities to the interventions provided by stroke liaison workers who are healthcare workers or volunteers who provide education, social support, and liaison with services. A 2010 Cochrane review (16 studies, 4759 participants) found no benefit in terms of perceived health, mood, activities or participation.^25^ As with EXTRAS, intervention participants were more satisfied with some aspects of service provision. Patients with mild to moderate disability (Barthel ADL score 15-19) who were supported by a stroke liaison worker were reported to have reduced death and disability (odds ratio, 0.62 [95% CI, 0.44 to 0.87]).

The LoTS (Longer Term Stroke) Care trial was published after the start of the EXTRAS trial.^21^ This large cluster RCT evaluated a system of care where stroke coordinators, who were community-based health care professionals with experience in stroke care, were trained to follow a structured assessment and treatment action plan working with patients and carers after hospital discharge. No improvement in outcomes was seen for patients or carers in the intervention group compared with usual care and health, and social care costs were similar between both randomization groups.

Self-management interventions seek to encourage people with chronic conditions to take an active part in their own care, and this was one of the components of EXTRAS. Approaches to self -management include: problem-solving; goal setting; decision making; self-monitoring; and coping with the condition. Self-management programmes seek to improve self-efficacy which is “the belief we have in our own abilities, specifically our ability to meet the challenges ahead of us, and complete a task successfully.”^26^ A 2016 Cochrane review of self-management programmes for people with stroke (14 studies, 1863 participants) reported that these programs improved quality of life (standardized mean difference random effects, 0.34 [95% CI, 0.05 to 0.62]) and improved self-efficacy (standardized mean difference random effects, 0.33 [95% CI, 0.04 to 0.61]), but they had no impact on activities of daily living or mood.^27^

The EXTRAS trial is one of the largest randomized controlled trials of a new community stroke service undertaken to date. All participants received stroke unit and ESD care before participating in the trial, in accordance with best practice. One of the strengths of the study is the completeness and quality of the data. Follow-up levels were high at all assessment points with low levels of missing data.

One potential criticism is that reviews were primarily undertaken by telephone. Telephone reviews were selected not only because they were more affordable, but also because they were likely to be less disruptive to a participant’s daily routine than a clinic visit. We did not use video or more sophisticated technology, as many participants would have not had access or experience in using them. As EXTRAS reviews were undertaken by telephone; we felt that it was important that they were undertaken by a stroke specialist with experience in community rehabilitation who knew the patient, who had expertise in goal setting, and detailed knowledge of local services. Reviewers were able to seek support and advice from other members of the ESD team. However, another potential criticism is that the study did not include a third randomization group to determine if nonspecialist support and reassurance could have resulted in similar improvements to mood and quality of life seen in the EXTRAS intervention group.

Participants in the control group received usual care post-ESD. Usual care is not standardized and, therefore, the care received by participants in the control group will have varied. Studies consistently report that patients with stroke receive very little longer term rehabilitation.^6,28,29^

Further research to develop and evaluate community services to meet the long-term and ongoing needs of stroke survivors, and carers is needed. In particular, longer term interventions to improve performance in extended activities of daily living. It will also be important to understand the mechanisms by which quality of life is improved, and costs are reduced by provision of preplanned intermittent longer term specialist review.

## Conclusions

EXTRAS did not significantly improve stroke survivors’ performance in extended activities of daily living. However, given the impact upon costs and QALYS, EXTRAS may be an affordable approach to providing ongoing specialist stroke care beyond ESD.

## Acknowledgments

We would like to thank the following for their contribution to this research project: patients and carers who participated; staff at the National Health Service study centers who facilitated the research including recruiting and treating participants; the National Institute for Health Research Stroke Research Network and National Institute for Health Research Clinical Research Network: Stroke; staff at Newcastle University who assisted with the delivery of the study.

## Sources of Funding

The National Institute for Health Research Health Technology Assessment Programme funded this trial. The views and opinions expressed here are those of the authors, and do not necessarily reflect those of the Health Technology Assessment programme, National Institute for Health Research, National Health Service or Department of Health.

## Disclosures

H. Rodgers declares fees from Bayer during this research project. G.A. Ford declares fees from AstroZeneca, Bayer, Medtronic, Pfizer, Pulse Therapeutics, Stryker and Amgen; grants from Daiichi Sankyo, Medtronic and Pfizer, during this research project. The other authors report no conflicts.

## Supplementary Material
