# Supplementary material for: Evaluation of an Extended Stroke Rehabilitation Service (EXTRAS): A Randomized Controlled Trial and Economic Analysis
Source: Stroke. 2019 Oct 22;50(12):3561–8. doi: 10.1161/STROKEAHA.119.024876 (PMC7597995; doi:10.1161/STROKEAHA.119.024876)

Supplemental Material

Supplemental Table I: Template for Intervention Description and Replication (TIDieR) checklist for EXTRAS

**1.** Brief name: *'provide the name or phrase that describes the intervention'*	Extended stroke rehabilitation service (EXTRAS)
**2.** Why: *'describe any rationale, theory, or goal of the elements essential to the intervention'*	The extended stroke rehabilitation service consisted of rehabilitation reviews conducted by a senior member of the Early Supported Discharge (ESD) team at 1, 3, 6, 12, and 18 months post discharge from routine ESD (trial randomisation). Each review consisted of a semi-structured interview to identify rehabilitation issues, goal setting and action planning.<br><br>The elements of this service are established rehabilitation practice and include: self-management; feedback; task practice; support; and referral to services if appropriate.
**3.** What materials: *'describe any physical or informational materials used in the intervention, including those provided to participants or used in intervention delivery or in training of intervention providers. Provide information on where the materials can be accessed'*	A study specific manual described how to conduct the reviews and included guidance on exploring rehabilitation needs, goal setting and appropriate interventions to meet a patient's needs.<br><br>Study specific paperwork was completed for each review and required documentation of identified needs, goals set and action plans made.<br><br>Staff delivering the reviews received study specific training.<br><br>Participants received an appointment card which documented review dates and also contained a short general checklist of rehabilitation issues to be covered in each review.<br><br>Following each review, participants received a summary of the interview and recommendations for rehabilitation by post.
**4.** What (procedures): *'describe each of the procedures, activities and/or processes used in the intervention, including any enabling support activities'*	EXTRAS reviews were intended to be conducted by telephone. However, if participation in a telephone interview was not possible, a home visit could be undertaken.<br><br>Each review consisted of:<br><br>1. Identification of rehabilitation needs. A semi-structured interview was conducted to determine the patient's progress, current rehabilitation needs and service provision. The interview addressed daily activities (mobility, personal care, meal times, domestic activities), social participation (work and volunteering, hobbies and interests, driving and transport) and

	wider issues (communication, memory and concentration, mood, medical issues, pain) which may be problematic for stroke survivors. The views of both the patient and carer (where appropriate) could be sought. The interview topics were based upon the literature about long term needs after stroke including the UK Stroke Survivor Needs Survey (McKevitt C, Fudge N, Redfern J, et al. Self-reported long-term needs after stroke. Stroke 2011; 42(5): 1398-40) and input from stroke survivors, carers and healthcare professionals.<br><br>2. Joint rehabilitation goal setting. From the identified progress and rehabilitation needs, up to five individual rehabilitation goals could be set by the patient (and carer) in collaboration with the senior ESD team member who conducted the review. The focus of joint goal setting was intended to be upon increasing participation in everyday activities. From the second review, progress towards goals set previously was assessed prior to further goal setting. Achievement of goals was recorded using a Goal Attainment Scale.<br><br>3. Action planning. The patient (and carer) agreed an action plan for each rehabilitation goal. Action plans were at the discretion of the reviewer and could include:<br>• Verbal advice and encouragement<br>• Discussion with the stroke team, rehabilitation team, primary care team, or social services currently involved in care<br>• Signposting to local activities, community organisations or voluntary services<br>• Referral to stroke services, rehabilitation services or primary care services for further assessment and treatment if appropriate according to local guidelines and/or service provision.
**5.** Who provided: *'for each category of intervention provider (for example, psychologist, nursing assistant) describe their expertise, background and any specific training given'*	Senior members of ESD teams delivered the reviews. The choice of ESD team staff member (e.g. physiotherapist, occupational therapist, nurse) to conduct each individual review was at the discretion of each participating centre.<br><br>On opening a study centre, all senior ESD staff taking part in delivery of the new service received face to face training from the EXTRAS co-ordinating centre team. As delivery of the intervention for this study was conducted for almost five years, inevitable staffing changes took place in ESD teams. New members of staff received cascade training from existing trained staff and/or further training visits from the EXTRAS co-ordinating centre were undertaken.
**6.** How: *'describe the modes of delivery (such as face to face or by some other mechanism, such as internet or telephone) of the intervention and whether it was provided individually or in a group'*	1:1 telephone interviews were intended. However, if participation in a telephone interview was not possible, a home visit could be undertaken.

**7.** Where: *'describe the type of location(s) where the intervention occurred'*	Telephone calls were made from ESD team facilities.
**8.** When and how much: *'describe the number of times the intervention was delivered and over what period of time including the number of sessions, their schedule, duration, intensity or dose'*	Five reviews were conducted over 18 months. These took place at 1, 3, 6, 12 and 18 months following discharge from routine ESD services. Up to two hours were available to complete each review. This included time to complete EXTRAS documentation, undertake any actions identified, and send a written summary to the patient and others involved in their care.
**9.** Tailoring: *If intervention was planned to be personalised or adapted, then describe what, why, when and how'*	Every review was personalised as individual needs were identified, goals set and actions planned.
**10.** Modifications: *'If intervention was modified during the course of the study, describe the changes what, why, when and how'*	No modifications were made to the intervention.
**11.** How well (planned): *'If intervention adherence or fidelity was assessed, describe how and by whom, and if any strategies were used to maintain or improve fidelity, describe them'*	Each review was documented onto study specific paperwork which asked for rehabilitation issues, goals set and action plans to be recorded. The paperwork for all five reviews was provided as a booklet to the person undertaking the reviews and at the start of each time point space was provided to document a reason if the review could not be undertaken. Data from the study paperwork was uploaded onto an online database and the study co-ordinating centre used this to send monthly reports to all participating teams highlighting overdue or missing reviews.
**12.** How well (actual): *'If intervention adherence or fidelity was assessed, describe the extent to which the intervention was delivered as planned'*	Summary information about delivery of the intervention is provided in the results section. Full details of the amount and content, including choice of goals, actions taken, and goal attainment of the EXTRAS intervention will be reported elsewhere.

Supplemental Table II: Comparison of patient experience of services at 12 and 24 months

	12 months			24 months		
**About the services received in the last 12 months** ***To what extent do you agree that***	**Patients satisfied/in agreement Intervention n (%)**	**Patients satisfied/in agreement Control n (%)**	**Difference in proportion of patients satisfied (Intervention-Control) (95% CI)**	**Patients satisfied/in agreement Intervention n (%)**	**Patients satisfied/in agreement Control n (%)**	**Difference in proportion of patients satisfied (Intervention-Control) (95% CI)**
Staff were welcoming and friendly	230 (99.1)	230 (97.5)	1.7 (-0.7 to 4.0)	114 (98.3)	123 (96.9)	1.4 (-2.4 to 5.3)
Staff treated you with dignity and respect	227 (98.3)	230 (97.1)	1.2 (-1.5 to 4.0)	117 (100.0)	122 (96.8)	**3.2 (0.1** to **6.2)**
Staff assessed your needs	219 (95.6)	229 (96.6)	-1.0 (-4.5 to 2.5)	113 (96.6)	114 (92.7)	3.9 (-1.8 to 9.6)
Staff met your needs	217 (93.9)	222 (93.7)	0.3 (-4.1 to 4.6)	114 (97.4)	111 (88.8)	**8.6 (2.4** to **14.9)**
You have been involved as much as you wanted to be in decisions about your care	211 (91.3)	213 (91.4)	-0.1 (-5.2 to 5.0)	110 (94.0)	108 (89.3)	4.8 (-2.2 to 11.8)
You were able to discuss your preferences, beliefs and concerns as part of your care	211 (93.0)	208 (92.4)	0.5 (-4.3 to 5.3)	106 (93.0)	112 (91.8)	1.2 (-5.6 to 7.9)
You were told who to contact if you had any worries or concerns	220 (94.4)	218 (93.6)	0.9 (-3.5 5.2)	110 (94.0)	116 (92.8)	1.2 (-5.0 to 7.5)
You were confident that the staff you saw had the right skills and knowledge to help you	223 (97.0)	223 (94.1)	2.9 (-0.9 to 6.6)	116 (97.5)	119 (94.4)	3.0 (-1.9 to 7.9)
You were treated fairly, regardless of your age, race, gender, belief, sexual orientation or disability	223 (96.5)	228 (96.6)	-0.1 (-3.4 to 3.2)	116 (99.2)	124 (96.9)	2.3 (-1.2 to 5.7)
You were given the information you wanted	220 (94.8)	219 (93.6)	1.2 (-3.0 to 5.5)	114 (96.6)	115 (92.0)	4.6 (-1.2 to 10.4)

	12 months			24 months		
**About the services received in the last 12 months** ***To what extent do you agree that***	**Patients satisfied/in agreement Intervention n (%)**	**Patients satisfied/in agreement Control n (%)**	**Difference in proportion of patients satisfied (Intervention-Control) (95% CI)**	**Patients satisfied/in agreement Intervention n (%)**	**Patients satisfied/in agreement Control n (%)**	**Difference in proportion of patients satisfied (Intervention-Control) (95% CI)**
You were able to see the same healthcare professional/team whenever possible	211 (92.5)	203 (87.1)	5.4 (-0.1 to 10.9)	103 (92.0)	107 (87.7)	4.3 (-3.4 to 12.0)
If you had important questions to ask, you got answers that you could understand	212 (93.0)	212 (95.5)	-2.5 (-6.8 to 1.8)	107 (93.0)	113 (92.6)	0.4 (-6.1 to 7.0)
If you needed more than one service, staff made sure they were well coordinated	196 (91.2)	189 (88.7)	2.4 (-3.3 to 8.1)	91 (91.9)	95 (91.4)	0.6 (-7.0 to 8.2)
If you needed more than one service, staff made sure that your care information was clearly and accurately shared	197 (92.1)	192 (89.7)	2.3 (-3.1 to 7.8)	91 (91.0)	94 (90.4)	0.6 (-7.4 to 8.6)
You were told who to contact if you had any ongoing health care needs	217 (93.1)	215 (91.5)	1.6 (-3.2 to 6.5)	110 (94.8)	115 (92.7)	2.1 (-4.0 to 8.2)
Overall, how satisfied are you with the services you received?	228 (95.4)	227 (92.3)	3.1 (-1.1 to 7.4)	208 (97.7)	189 (87.5)	**10.2 (5.3** to **15.0)**
In the last 12 months, have you had enough help with speaking difficulties from the NHS?	79 (87.8)	89 (84.8)	3.0 (-6.6 to 12.7)	29 (69.1)	35 (63.6)	5.4 (-13.5 to 24.3)
In the last 12 months, have you had enough treatment to help improve your mobility	167 (82.7)	166 (76.2)	6.5 (-1.2 to 14.2)	95 (67.4)	81 (48.5)	**18.9 (8.0** to **29.7)**

	**12 months**			**24 months**		
**About the services received in the last 12 months** ***To what extent do you agree that***	**Patients satisfied/in agreement Intervention n (%)**	**Patients satisfied/in agreement Control n (%)**	**Difference in proportion of patients satisfied (Intervention-Control) (95% CI)**	**Patients satisfied/in agreement Intervention n (%)**	**Patients satisfied/in agreement Control n (%)**	**Difference in proportion of patients satisfied (Intervention-Control) (95% CI)**
from the NHS?						
In the last 12 months, have you had enough help with emotional problems from the NHS?	80 (70.8)	112 (76.7)	-5.9(-16.7 to 4.9)	52 (71.2)	59 (62.1)	9.1 (-5.1 to 23.4)

Supplemental Figure IA: Association between pre-stroke health status and effectiveness of EXTRAS

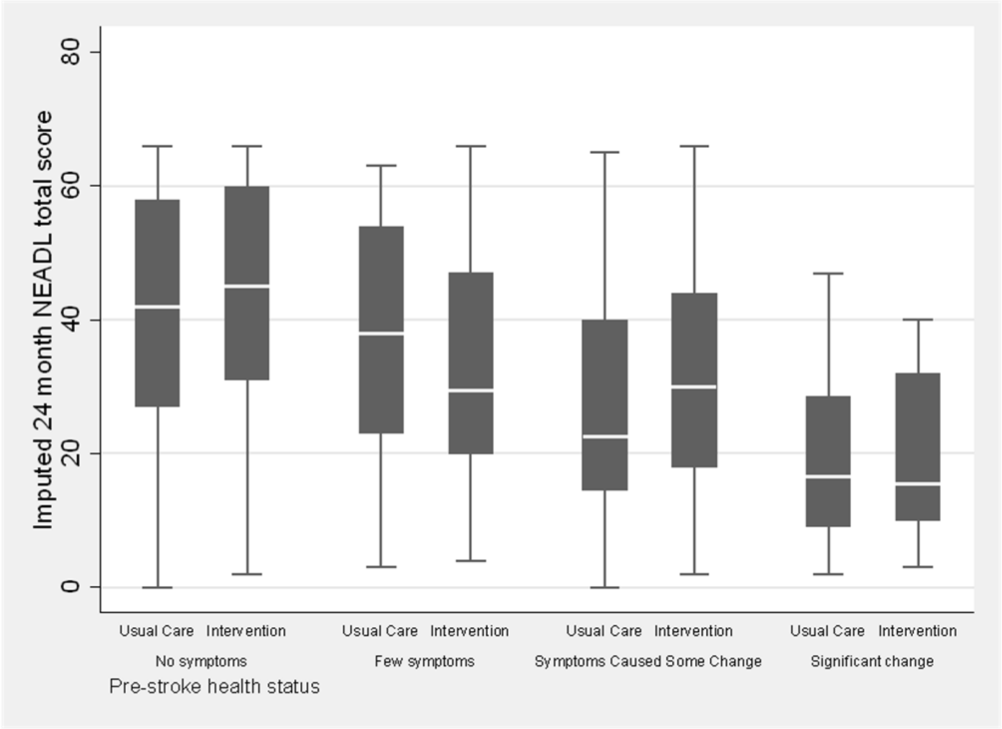

Supplemental Figure IB: Association between severity of activity limitation and effectiveness of EXTRAS

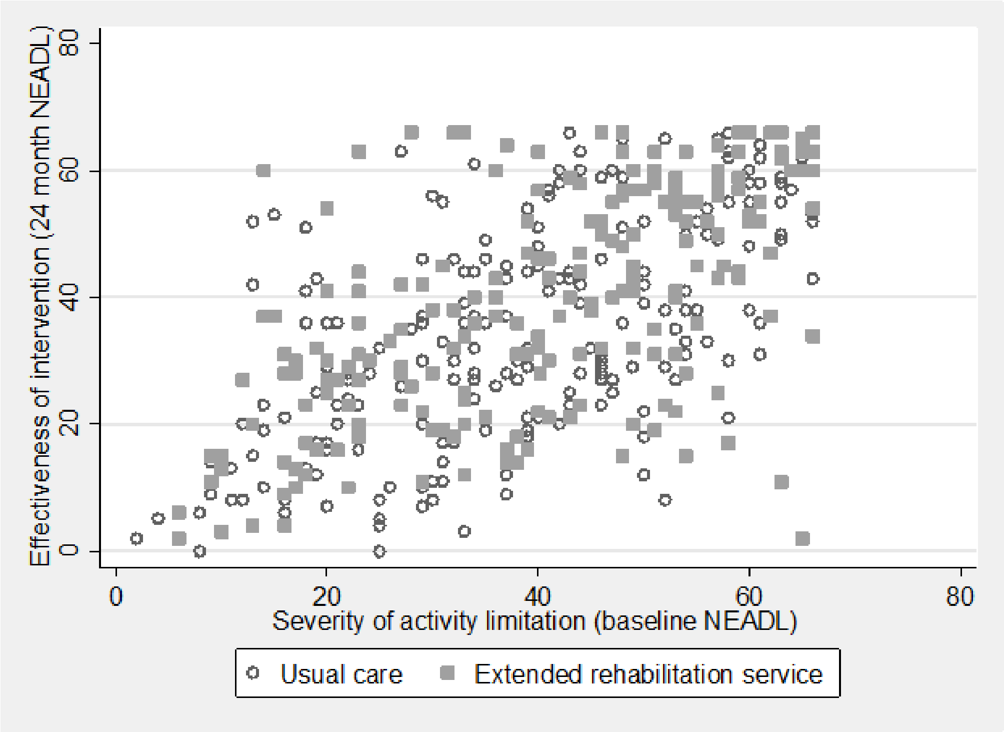

Supplemental Figure IC: Association between length of time of organised care and effectiveness of EXTRAS

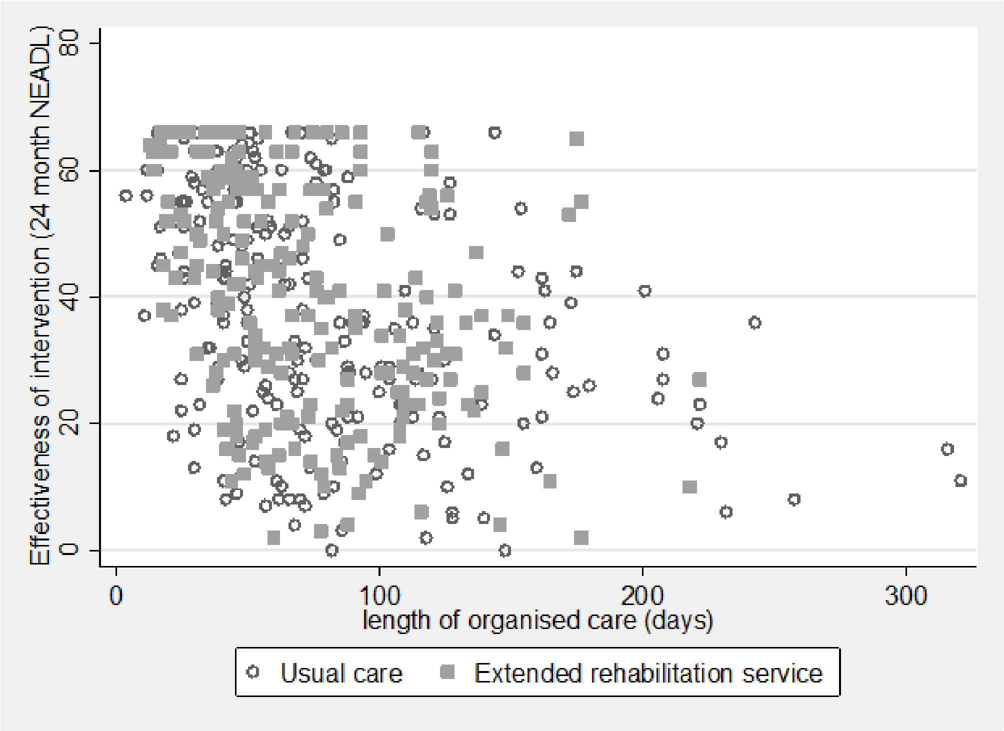

Supplement: Supplementary file 1 [file str-50-3561-s001.pdf]
